# Contrast Sensitivity, Visual Acuity, and Cognition in Older Adults for the Healthy Aging Initiative

**DOI:** 10.1093/geroni/igaf122.3229

**Published:** 2025-12-31

**Authors:** Molly Quigley, John Woolley, Talia Gilfix, Heather Andrews, Yael Koren, On-Yee Lo, Davide Cappon, Alvaro Pascual-Leone

**Affiliations:** Hebrew SeniorLife, Boston, Massachusetts, United States; Hebrew SeniorLife, Boston, Massachusetts, United States; Hebrew SeniorLife, Boston, Massachusetts, United States; Harvard T.H. Chan School of Public Health, Boston, Massachusetts, United States; Hebrew SeniorLife, Boston, Massachusetts, United States; Harvard Medical School/ Marcus, Hebrew SeniorLife, Boston, Massachusetts, United States; Hebrew SeniorLife & Harvard Medical School, Boston, Massachusetts, United States; Hebrew SeniorLife & Harvard Medical School, Boston, Massachusetts, United States

## Abstract

Visual dysfunction in older adults can be associated with cognitive impairment. Cognitive assessments are not regularly utilized by many clinicians as they are time-consuming, susceptible to learning effects, and require complex instructions. Assessing visual function through contrast sensitivity and visual acuity tests may help predict cognitive decline and aid clinicians in its early detection while minimizing implementation barriers. We analyzed data from the first 148 participants of the Healthy Aging Initiative (HAI), a longitudinal observational study designed to track health changes over time in residents living independently in Hebrew SeniorLife Senior Housing Communities. We assessed visual acuity (Rosenbaum Near-Vision Card), contrast sensitivity (Pelli-Robson Contrast Sensitivity Chart), and cognitive function (Mini-Cog, Trail Making Test Part B, and Rey-Auditory Verbal Learning Test [RAVLT]). Cognitive measures were adjusted for demographic norms. We analyzed cross-sectional associations between visual and cognitive measures using Pearson correlations. Contrast sensitivity was significantly associated with both Mini-Cog and RAVLT (p = 0.019; p = 0.006, respectively). A trend was observed between visual acuity and Mini-Cog scores (p = 0.092). No other significant associations were identified. Our results align with existing literature indicating that contrast sensitivity is significantly associated with cognition. The trend between visual acuity and cognition also corresponds with existing literature supporting its value. Annual wellness exams for older adults should include assessments of visual domains, especially in senior housing communities where resident health is closely monitored. Contrast sensitivity, which is associated with memory deficits, may be particularly valuable as a brief and low-cost method for early detection of Alzheimer’s disease and related dementias.

